# The Effect of Oral Pure Branched-Chain Amino Acid Supplementation on Exercise Performance and Body Composition: A Systematic Review

**DOI:** 10.7759/cureus.96017

**Published:** 2025-11-03

**Authors:** Maxim Julea, Sohag N Saleh

**Affiliations:** 1 Pharmacology, Imperial College London, London, GBR; 2 Critical Care Medicine, Doncaster and Bassetlaw Teaching Hospitals, Doncaster, GBR

**Keywords:** body composition, branched-chain amino acid, exercise, nutrition, performance, supplements

## Abstract

Branched-chain amino acids (BCAAs) are an increasingly popular sports supplement used by athletes as they are advertised to increase endurance, muscle recovery, lean muscle mass, and decrease muscle soreness and fatigue. However, the mechanisms behind BCAAs are not well-established, and previous studies have had mostly equivocal results; thus, the possible performance-enhancing effect of BCAAs is controversial. The aim of this systematic review was to evaluate the current literature on the effect of pure BCAA supplementation on exercise performance and body composition. Randomized controlled trials with healthy participants >18 years old were included. The intervention was pure combined BCAA supplements, and the comparator was a matched control. Studies with outcomes related to exercise performance and/or body composition were eligible. PubMed, Ovid Medline, Scopus, Web of Science, and Google Scholar were searched until 15/08/2025. We assessed selection, attrition, performance, detection, attrition, reporting and other biases from low to high risk based on the Cochrane Handbook for Systematic Reviews. Twenty-two studies were included with 511 participants in total. Eleven studies had trained athletes, whereas 11 articles had untrained participants. Daily doses ranged from 1.5g to 82g. The age varied from 18 to 71.7 years, whereas the study length varied from one day to six months. One study found significant differences in body composition after BCAA supplementation. There were 13 studies with significant findings for various exercise performance outcomes such as endurance, strength, muscle recovery, speed, and fitness. The results moderately support BCAA reducing muscle soreness; however, there is inconsistent evidence to support BCAAs having an ergogenic effect on strength and endurance. Due to unclear/high risk of bias in several studies, no meta-analysis was performed. In conclusion, BCAA supplementation may reduce muscle soreness post-exercise, but evidence for improvements in strength, endurance, and body composition remains inconsistent. However, future studies should consider improving certain limitations such as implementing incremental doses, strict diets, and enrolling more participants with diverse backgrounds.

## Introduction and background

Prevalence of branched-chain amino acid (BCAA) supplements

Sports supplements are increasing in popularity as more people take them to improve performance [1]. One of these supplements is BCAAs, which are advertised as ergogenic aids that increase endurance, muscle recovery, and decrease muscle soreness and fatigue [2]. Moreover, supplement companies claim that BCAAs enhance body composition by increasing/preserving lean muscle mass [2]. In a 2017 national survey of about 23028 US college athletes, 13.2% reported taking amino acid supplements for sports performance [3]. In 2022, the global BCAA market was valued at approximately USD 1.45 billion and is projected to reach USD 2.6 billion by 2032 [4]. Despite the advertising claims, previous studies have shown inconsistent effects of BCAAs on performance and body composition [5-7].

BCAA physiology

**Figure 1:**
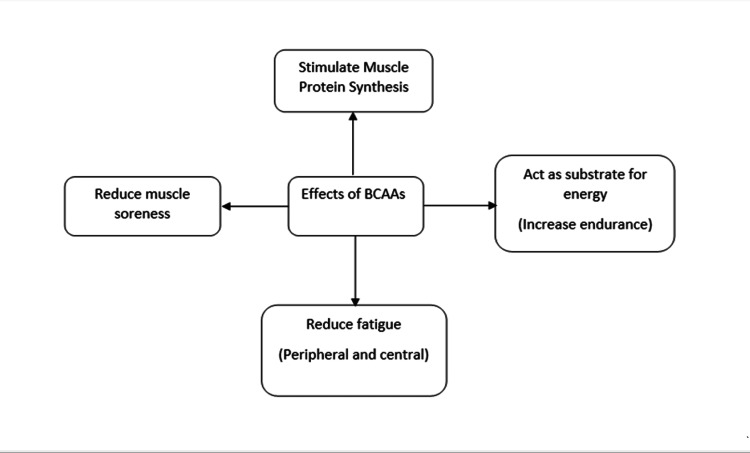
Mind map of the main hypothesized physiological effects of BCAAs. Created by the author BCAA: Branched-chain amino acid

BCAAs (leucine, isoleucine, and valine) are part of the essential amino acid (EAA) category, which cannot be synthesized by the human body and hence, need to be sourced from the diet [8]. BCAAs are abundant in skeletal muscle as they are the main component of the structural proteins in muscles and play various nutritional and functional roles [9]. BCAAs can be used as an energy substrate during exercise [10,11], function as a signaling factor during protein synthesis [10,12], inhibit protein catabolism via the ubiquitin-proteosome pathway [13], stimulate myotube proliferation [12] and decrease inflammatory cytokine release and oxidative stress [11,14] (see Figure 1 for summary). However, there is limited research on some of the effects of BCAAs, such as decreasing fatigue [15] or the physiology of reducing muscle soreness [16].

Exercise-induced muscle damage

It is well established that eccentric exercise causes muscle damage and pain [17]. Exercise-induced muscle damage (EIMD) is believed to occur due to degradation of myofibrillar proteins (proteolysis) [17]. Furthermore, proteolysis leads to increased myofiber proteins (e.g. creatine kinase and myoglobin) in the blood [18]. EIMD symptoms include increased muscle soreness, pain and reduced range of motion and muscle function [19]. These symptoms reduce performance and recovery, which may prevent repeated exercise sessions which are needed for adaptations to exercise, such as enhanced exercise tolerance or muscle hypertrophy [18]. Seeking a solution for EIMD, adequate nutrition was shown to limit muscle protein breakdown [20]. Consequently, nutritional interventions such as protein and amino acid supplements have been studied to improve performance by reducing the deleterious effects of exercise [21,22].

Muscle soreness

BCAAs may increase the rate of recovery after exercise by activating the mechanistic target of rapamycin complex 1 (mTORC1) and the nuclear factor kappa B (NFkB) signaling pathways in muscle cells [11], lymphocytes [23], and macrophages [11,23]. Consequently, by increasing recovery, BCAAs are hypothesized to reduce the symptoms of delayed-onset muscle soreness (DOMS) that occur after exercise. Moreover, activation of certain neurotrophin pathways (Nerve Growth Factor- NGF and cyclooxygenase enzyme- COX-2) contributes to the development of DOMS [24,25]. As BCAAs have been shown to prevent the upregulation of neurotrophin pathways, this is another way BCAAs are believed to aid muscle recovery [9].

Muscle synthesis

Muscle tissues are composed of two protein types, i.e. actin and myosin [26]. Actin and myosin consist of leucine, isoleucine, and valine [26]. Consequently, replenishing BCAAs increases the raw materials available for muscle synthesis. It has been shown that BCAA-enriched supplements (such as whey protein or essential amino acids), given post-exercise, augment muscle synthesis and aid recovery [27,28]. BCAA supplements, in tandem with exercise, were shown to help muscle synthesis by stimulating mTOR and p70-S6 kinase, both of which are key regulators in cell growth, transcription, and protein synthesis [29].

Energy substrate

BCAAs are primarily oxidized in skeletal muscle and can contribute as an energy substrate during exercise [30]. Isoleucine and valine may increase oxaloacetate levels which may increase free fatty acid oxidation, especially in a glycogen-depleted state [31]. Consequently, BCAAs may increase endurance by providing extra energy in glycogen-depleted states e.g. during fasting or prolonged exercise [31].

Fatigue

BCAAs are also hypothesized to decrease fatigue by improving fatigue substances levels in the blood. Previous studies found that fatigue can have peripheral or central causes [32]. BCAAs may help reduce peripheral fatigue substances such as lactate and ammonia or central fatigue substances like serotonin (5-HT) [33]. Brain 5-HT increases during exercise; thus, it is believed to be a cause of central fatigue [33,34]. Free tryptophan increases uptake of serotonin in the brain [34]. As BCAAs compete for the same carrier as tryptophan, increased blood levels of tryptophan are believed to decrease serotonin uptake in the brain and hence reduce central fatigue [33,34]. Consequently, by reducing fatigue, athletes may be able to perform exercise for longer bouts.

Body composition

The mechanism behind how BCAAs may enhance body composition is not clear; however, it is believed to be a mixture of BCAAs stimulating muscle protein synthesis [35], preventing muscle catabolism [36], and promoting fat oxidation [31] as an alternative energy substrate. Consequently, these three processes combined are thought to aid athletes increase or preserve lean muscle mass.

Formulations of BCAAs

The effects of BCAAs have been studied in different formulations; however, in this paper, we investigated the combined formulation which is their most common mode of administration [37]. Leucine monotherapy has been studied, and findings indicate that leucine alone can stimulate an anabolic effect [38], whereas there is no evidence for such an effect for isoleucine or valine [8]. Thus, it may be expected that leucine alone would be more effective than the combined form of all BCAAs. However, supplementation with leucine alone has its limitations. Firstly, leucine administered alone would limit the extent of muscle protein synthesis due to the lack of availability of other essential amino acids [8]. Secondly, leucine administration stimulates the oxidation of all other amino acids; thus, it may lead to depletion of isoleucine and valine. Consequently, it is believed that co-administration of leucine with isoleucine and valine is the most effective formulation [8]. On the other side, it has been proposed that the combined form of BCAAs, although preferred, could limit leucine effects due to competition between amino acids for transport into muscle fibres [37]. Moreover, supplements with BCAA alone may not be as effective at controlling muscle protein turnover compared to supplements with more essential amino acids [8,37].

Some independent studies have studied oral BCAA supplementation in humans and found BCAAs to stimulate muscle protein synthesis, reduce muscle protein breakdown, EIMD and fatigue with doses ranging from 77mg/kg to 3g/kg [37]. The most used BCAA ratio is 2:1:1 leucine, isoleucine, and valine, respectively, as BCAA supplementation is safest if in proportions close to mammal body protein [30].

Aims and hypotheses

Supplements with BCAAs commonly contain other ingredients such as caffeine, creatine, carbohydrates, or vitamins [6,15,39]. Some of these ingredients are proven ergogenic aids [40,41]. Thus they may have acted as confounders in previous studies investigating the effect of BCAAs on exercise performance. In addition, some of the effects in humans that are advertised by producers are hypothesized from cellular and animal models [6]. Also, previous studies done with humans have mostly equivocal findings [5-7]. Thus, the aim of this systematic review was to investigate the effects of pure combined BCAA supplementation on exercise performance and body composition by synthesizing and updating the current randomized controlled trial evidence. Both acute and chronic supplementation protocols were considered, and the review evaluates outcomes across multiple performance domains, including endurance, strength, and muscle recovery.

Based on the previous literature, our null hypothesis was that BCAA supplementation would have no effect on performance or body composition. Contrastingly, our alternative hypothesis was that BCAA supplementation would increase exercise performance and fat-free muscle mass.

## Review

Methods

This systematic review followed the guidelines of the Preferred Reporting Items for Systematic Reviews and Meta-Analyses (PRISMA) [42].

Literature Search

A search for articles investigating the effect of BCAA supplementation on exercise performance and body composition was performed using the following international databases: PubMed, Ovid Medline, Scopus, and Web of Science. Google Scholar was used as a supplementary search engine. The search was carried out for all time until 15/08/2025. The search was limited to studies in English and Portuguese as these were the languages accessible to the reviewer and included all studies identified as relevant during preliminary screening. The single study published in Portuguese was translated into English using Google Translate and manually checked for accuracy and terminology consistency by the primary reviewer. Furthermore, the following search strategy was used ("branched-chain amino acid*" OR "bcaa") AND ("exercise" OR "exercise tolerance" OR "sport*" OR "endurance" OR "resistance" OR "train*" OR "athlet*" OR "stamina" OR "performance" or "fatigue" or "fitness" or "muscle damage" or "muscle soreness"). Google Scholar was searched using a refined version of the same core search strategy, and the first 210 records were screened, consistent with prior systematic review practice recognizing the limited relevance of later-ranked results. The detailed search strategies for all databases are provided in the Appendices (see Table 4).

Inclusion and Exclusion Criteria

The studies were selected based on Population, Intervention, Comparator, Outcomes and Study design (PICOS system). To observe the physiological effect of BCAAs, the population was composed of healthy individuals >18 years old without chronic diseases. A minimum of five participants per study arm was required. This threshold was selected to ensure adequate data for between-group comparison and to exclude very small case-series and aligns with current concerns regarding underpowered trials in sports and exercise science [43,44]. Moreover, the intervention was defined as a combined BCAA supplement (only leucine, isoleucine, and valine). In addition, studies had to include at least one group with a pure BCAA to observe isolated effects; thus, any studies with extra ingredients were excluded. The comparator was a matched control. The outcomes included were any measure of exercise performance and body composition. The study design was restricted to randomized controlled trials. Furthermore, any dissertations, theses, partially published articles, or articles with only abstracts were excluded.

Data Collection and Analysis

**Figure 2:**
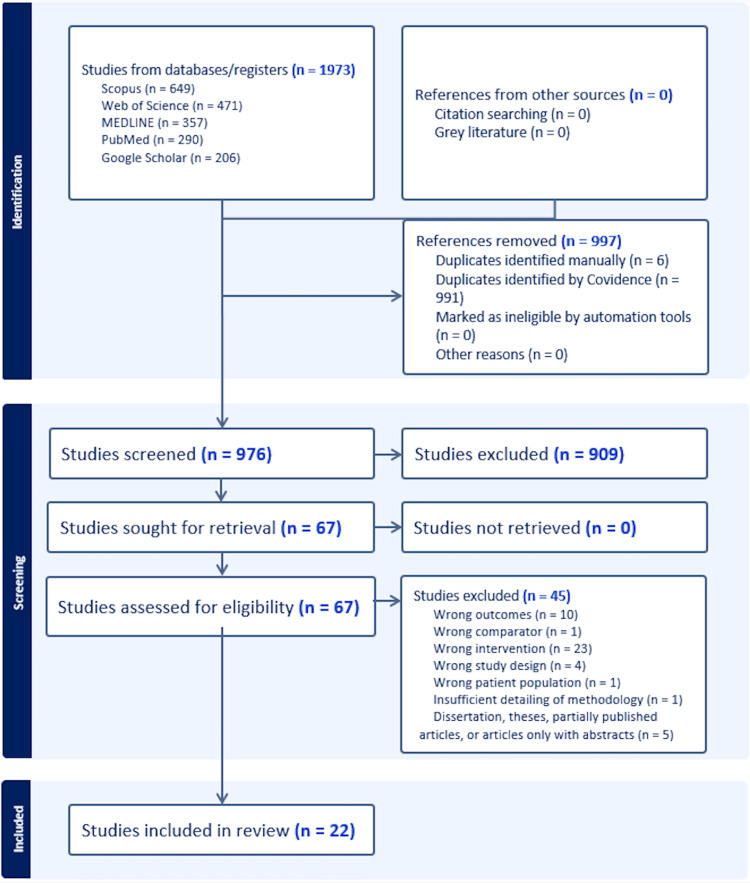
PRISMA flowchart indicating the distinct levels of screening, reasons for excluding articles and numbers of included/excluded articles at each stage PRISMA: Preferred Reporting Items for Systematic Reviews and Meta-Analyses

All records identified through database searches were imported into Covidence (Veritas Health Innovation, Melbourne, Australia) for screening. Duplicates were removed automatically within the platform and verified manually to ensure accuracy. Titles and abstracts were then screened to remove irrelevant studies, reviews, animal experiments, and non-randomized designs. Full texts of potentially eligible articles were retrieved and assessed against predefined inclusion and exclusion criteria. Any studies not meeting these criteria were excluded, and reasons for exclusion were documented (see Figure 2).

Risk of Bias

Studies were assessed in RevMan 5 for selection bias, performance bias, detection bias, attrition bias, reporting bias and other biases (e.g. sponsors, uncontrolled variables) based on the Cochrane Handbook for Systematic Reviews [43]. Studies were classified as unclear risk (if there was insufficient information to quantify risk), high risk (factors that could strongly influence objectivity of findings) or low risk (all reasonable information and controlled variables are clearly presented and accounted for). The Cochrane Handbook [43] suggests that meta-analyses should not be performed when there is risk of bias in all/some studies; thus, as all RCTs included had instances of unclear/high risk, we chose not to perform a meta-analysis to avoid misleading results.

Results

Studies

After the initial database search, 1973 articles were identified. Following removal of duplicates, 976 articles were screened by title and abstract. Of these, 909 articles were excluded as irrelevant, reviews, animal studies, or non-randomized trials; 67 full text articles were then assessed for eligibility, of which 45 were excluded for not meeting the inclusion criteria (e.g. mixed supplements, wrong population, or non-English/Portuguese text). Consequently, 22 studies met all inclusion criteria and were included in the final analysis (Figure 2). The search covered all years up to 15 August 2025, and the publication years of included articles ranged from 2004 to 2025. Study durations varied from one day to six months. Of the 22 included studies, 21 were published in English and one in Portuguese.

Participants

The included studies were randomized controlled trials. A total of 511 people participated in these studies. The number of participants varied from 5 to 100 with a sample mean of 23.2 +/-19.2 participants. The ages varied from 18 to 71.7 years. Eighteen studies were done only on men, one was done only on women, and three included both women and men. Eleven studies included untrained participants (sedentary or healthy/active), whereas 11 included trained participants (athletes or “resistance-trained”) (see Table 1).

**Table 1:** Randomized controlled trials included (n=22) with details of study design, age and numbers of participants, exercise protocol, diet, investigated outcomes and study length RCT: randomized controlled trial; CK: creatine kinase; LDH: lactate dehydrogenase; PMS: perceived muscle soreness; RM: maximum repetitions; MVC: maximal voluntary contraction; HR: heart rate; RPE: rating of perceived exertion; 1RM: maximum weight displaced for one repetition; CRP: C-reactive protein; CMJ: countermovement jump

Author and Year	Type of Study and Sample Size	Age Range	Gender	Type of Exercise	Diet	Investigated Outcomes	Length and Follow-Up	
Aminiaghdam (2012) [45]	Double-blind RCT with soccer players (n=30)	20.2±0.7	Male	Resistance training	Maintain usual diet	CK, LDH, PMS	Nine days	
Areces (2014) [46]	Double-blind, RCT with experienced runners (n=46)	41.4±7.4	Male & Female	Aerobic exercise (running)	Maintain usual diet	Vertical jump, handgrip, lower limb strength and power, running speed, PMS, urine myoglobin, urine pH, urine protein	Seven days	
Asjodi (2018) [47]	RCT with untrained participants (n=50)	22.2±2.3	Male	Resistance training	Maintain usual diet	CK, LDH, RM, PMS	72h	
Bagheri (2021) [48]	Double-blind, RCT with postmenopausal women (n=30)	56±3.7	Female	Resistance training	Maintain usual diet	Body composition, strength, myokines and IGF-1	Eight weeks	
Dorrell (2016) [49]	Single-blind, counterbalanced RCT with resistance-trained participants (n=5)	21.8±0.8	Male	Mixture of Plyometric, Aerobic and resistance training	Maintain usual diet	Countermovement (CMJ) and squat jump (SJ), peak (PP) and mean (MP) power, Medicine Ball Throw distance, PMS	One day	
Fouré (2016) [50]	Double-blind, RCT with young healthy participants (n=26)	22.5±1.6	Male	Neuromuscular stimulation	Maintain usual diet	CK, phosphocreatine (PCr), inorganic phosphate (Pi), MVC, power, PMS, alanine, citrulline, tyrosine, phenylalanine, glycine, histidine, leucine, isoleucine, valine	Eleven days	
Gee (2016) [51]	Single-blind counterbalanced RCT with resistance-trained participants (n=11)	24.7±5.9	Male	Resistance training	Maintain usual diet	PMS, countermovement jump, seated shot-put throw	One day	
Greer (2011) [52]	Double-blind, cross-over RCT with sedentary participants (n=9)	21.6±3.2	Male	Aerobic exercise (Cycling)	Maintain usual diet	Total distance performed, perceived exertion, respiratory exchange ratio	One day, with eight weeks between supplementation protocols	
Gualano (2011) [31]	Double-blind cross-over RCT with healthy participants (n=7)	24±2	Male	Aerobic exercise(running)	Maintain usual diet	Time to exhaustion, respiratory exchange ratio (RER), plasma glucose, free fatty acids (FFA), blood ketones and lactate	Three days	
Howatson (2012) [53]	Double-blind RCT with rugby and football competitors (n=12)	23±2	Male	Plyometric exercise (drop jumps)	Maintain usual diet	CK, PMS, MVC, vertical jump and thigh and calf circumference.	Twelve days	
Luan (2025) [54]	Double-blind RCT with young healthy active college students (n=11)	21±1	Male	Aerobic exercise (cycling)	Logged dietary intake- same plan was replicated for all trials	Fat/CHO oxidation, cycling efficiency, fatigue (VAS), RPE, HR, insulin, blood ammonia, time to exhaustion	Three days	
Manaf (2021) [55]	Double-blind counterbalanced crossover RCT with recreationally active participants (n=18)	24.7±4.8	Male	Aerobic exercise (cycling)	Controlled dietary intake- same plan was replicated for all trials	Rating of perceived exertion, power, cadence, HR, MVC, muscle voluntary activation level and electrically evoked torque using single and doublet stimulations	One day	
Martín-Martínez (2020) [56]	Double-blind RCT with professional volleyball players (n=12)	24.6±3.8	Male	Plyometric exercise (vertical jumps)	Maintain usual diet	Countermovement vertical jump	Seven days	
Meng (2025) [57]	Double-blind crossover RCT with untrained males (n=24)	23.1±2.2	Male	Resistance training and plyometric exercise (vertical jumps)	Maintain usual diet (logged calories and protein for 3 days before trial)	VAS (soreness), CMJ, IL-6, CRP, CK, blood lactate (30 min, 24h, 48h post)	One day (doses 1x Pre & 1x Post) with seven-day washout and crossover	
Mor (2022) [58]	Single-blind RCT with football players (n=24)	20.5±2.3	Male	Aerobic exercise	Maintain usual diet	Running anaerobic speed test, ball speed measurement, blood lactate, HR, power	Seven days	
Muscella (2024) [59]	Double-blind RCT with resistance-trained candidates (n=100)	35.9±10.0	Male & Female (50/50)	Resistance training	Maintain usual diet-logged food intake	Body composition (lean/fat/muscle), 1RM (squat, bench, deadlift), DOMS (VAS), fatigue (ROF scale)	Six months	
Robbins (2025) [60]	Pilot double-blind RCT with older adults (n=20)	70.5±1.2	Male and female (63% F/37%M)	Supervised aerobic + resistance training, 3x/week	Diet monitored and adjusted based on Healthy Eating index	Handgrip strength, chair stands, gait speed, VO2 max, 400m walk; CES-D, FAS, ISI, VAS (QoL, fatigue, pain)	Eight weeks	
Shenoy (2017) [61]	Stratified double-blind RCT with road cyclists (n=20)	20±1.2	Male	Plyometric exercise (drop jumps)	Maintain usual diet	CK, High sensitivity C- reactive protein (hs-crp), and myeloperoxidase (MPO), isometric knee muscle strength, PMS, aerobic capacity (VO2max), HR	28 days	
Smith (2018) [62]	Double-blind RCT with resistance-trained participants (n=13)	23±3.8	Male	Resistance training	Maintain usual diet	Cortisol, glucose, insulin, resistance exercise performance	Seven days with blood tests 60 min after exercise	
Uchida (2008) [63]	Double-blind, cross-over RCT with healthy participants from the Brazilian Army (n=17)	22±2	Male	Aerobic exercise (running)	Controlled diet with 16% protein	Lactate, ammonia, total distance performed, perceived exertion, time to exhaustion	One day, with seven days between supplementation protocols	
VanDusseldorp (2018) [64]	Double-blind, RCT with active resistance trained participants (n=20)	22.3±1.5	Male	Resistance training	Controlled diet with protein 1.2 g/kg body weight	CK, vertical jump, MVC, jump squat peak power, PMS	Eight days	
Watson (2004) [65]	Double-blind RCT with recreationally active males (n=8)	28.5±8.2	Male	Aerobic exercise (cycling in warm environment i.e. 30°C & 38% humidity	Standardized diet and activity for 48h pre-test	Time to exhaustion, HR, rectal/skin temp, RPE, thermal comfort, blood glucose & lactate, expired air parameters	One day testing (one week washout and crossover)	

Type of Exercise

Types of exercise included resistance exercise (seven studies) and aerobic exercise (eight studies), plyometric exercise (three studies), mixture/multiple (three studies) and neuromuscular stimulation (one study) (see Table 1).

Supplementation

The BCAA doses included either fixed doses or individual doses (calculated per body mass) (Table 2). Overall, the daily dose varied from 1.5 to 82g. The control was either a carbohydrate or an artificial sweetener. Length and time of administration varied from a single dose to multiple doses per day for up to six months. All included trials administered pure BCAA supplements without additional amino acids, carbohydrates, or other ergogenic additives.

**Table 2:** Supplementation protocol of included randomized controlled trials (n=22)

Author/Year	Composition Leu/Iso/Val	Total Daily Amount (g)	Individual Dose (mg/kg of Body Weight)	Supplementation Length	Matched Control	Supplementation Details
Aminiaghdam (2012) [45]	2:1:1	~15.1g base o r ~44.8g or ~81.9g on exercise days	68 mg/kg +200mg/kg or 450 mg/kg 30 min before or after exercise	9 days	Dextrin	3X/day with extra 200 mg/kg or 450 mg/kg before and after exercise
Areces (2014) [46]	2:1:1	5g	-	7 days	Dextrose: Cellulose	Once daily
Asjodi (2018) [47]	1:1:1	~1.5g	10 mg/kg	1 day	Maltodextrin(30mg/kg)	2X/ day split 30 min before and immediately after exercise
Bagheri (2021) [48]	2:1:1	9g	-	3X/week for 8 weeks	Guar gum	2X/ day split 30 min before and immediately after exercise
Dorrell (2016) [49]	2:1:1	12g or 36g (daily)	-	1 day	Artificial sweetener	6g or 18 g 20 min before and after exercise
Fouré (2016) [50]	2:1:1	~7g	100mg/kg	5 days	Cellulose	3 doses on Day 1, then 1 dose/day for Days 2-5
Gee (2016) [51]	2:1:1	20g	-	1 day	Artificial sweetener	2X/ day split before and after exercise
Greer (2011) [52]	2:1:1	48.6g	-	1 day	Artificial sweetener	2X/ day split before and after exercise
Gualano (2011) [31]	-	-	300mg/kg/day	3 days	Maltodextrin	1X/ day before exercise
Howatson (2012) [53]	2:1:1	20g	-	12 days	Artificial sweetener	2X/ day split 10g with an additional 20g before and after exercise
Luan (2024) [54]	2:1:1	-	0.2 g/kg body weight/each time	3 days(pre-ex) + 1 dose on exercise day	Isocaloric starch	2X/ day for three days and then once in the morning of exercise day
Manaf (2021) [55]	2:1:1	9.4g	0.084 mg/kg before + 0.056 mg/kg*h during exercise	1 day	Non-caloric cordial	-
Martín-Martínez (2020) [56]	2:1:1	7g	--	3 days during a week	Watermelon flavored drink	7g/ day Monday, Wednesday, and Friday
Meng (2025) [57]	2:1:1	-	40mg/kg	1 day (2 doses)	Maltodextrin	1X Supplement 30 min pre- exercise 1x supplement immediately post-exercise 7-day washout then crossover
Mor (2022) [58]	2:1:1	5g	-	7 days	Bran	2X/ day split 30 min before and 1h after exercise
Muscella (2024) [59]	2:1:1	7-10g	100 mg/kg body weight	6 months	Undefined placebo	Daily – on exercise days, immediately after exercise, non -exercise to consume in morning, supplement contained lecithin
Robbins (2025) [60]	2:1:1	5g	100 mg/kg/day	8 weeks	Maltodextrin	Acute intervention- one dose pre-ex on first day- then measured; long-term intervention- eight weeks daily supplementation followed by re-measurement
Shenoy (2017) [61]	2:1:1	20g	-	28 days	Artificial sweetener	2X/ day split 10g
Smith (2018) [62]	2:1:1	7.5g	-	1 day	Artificially flavored water	Six doses split before warm-up, after warm-up and after exercise
Uchida (2008) [63]	4:3:3	-	77mg/kg	1 day	Maltodextrin	2X/ day split 45 min before and 20 min before
VanDusseldorp (2018) [64]	3:1:2	-	220mg/kg/day	8 days	Maltodextrin	2X/ day split one in the morning and one in the evening
Watson (2004) [65]	2:1:1	12g base+ 5.4-18g	-	1 day	Sugar-free fruit drink	12g during 2h before exercise then varying increments during exercise (until failure). Increments based on exercise length. Seven-day washout, then crossover

Risk of Bias

**Figure 3:**
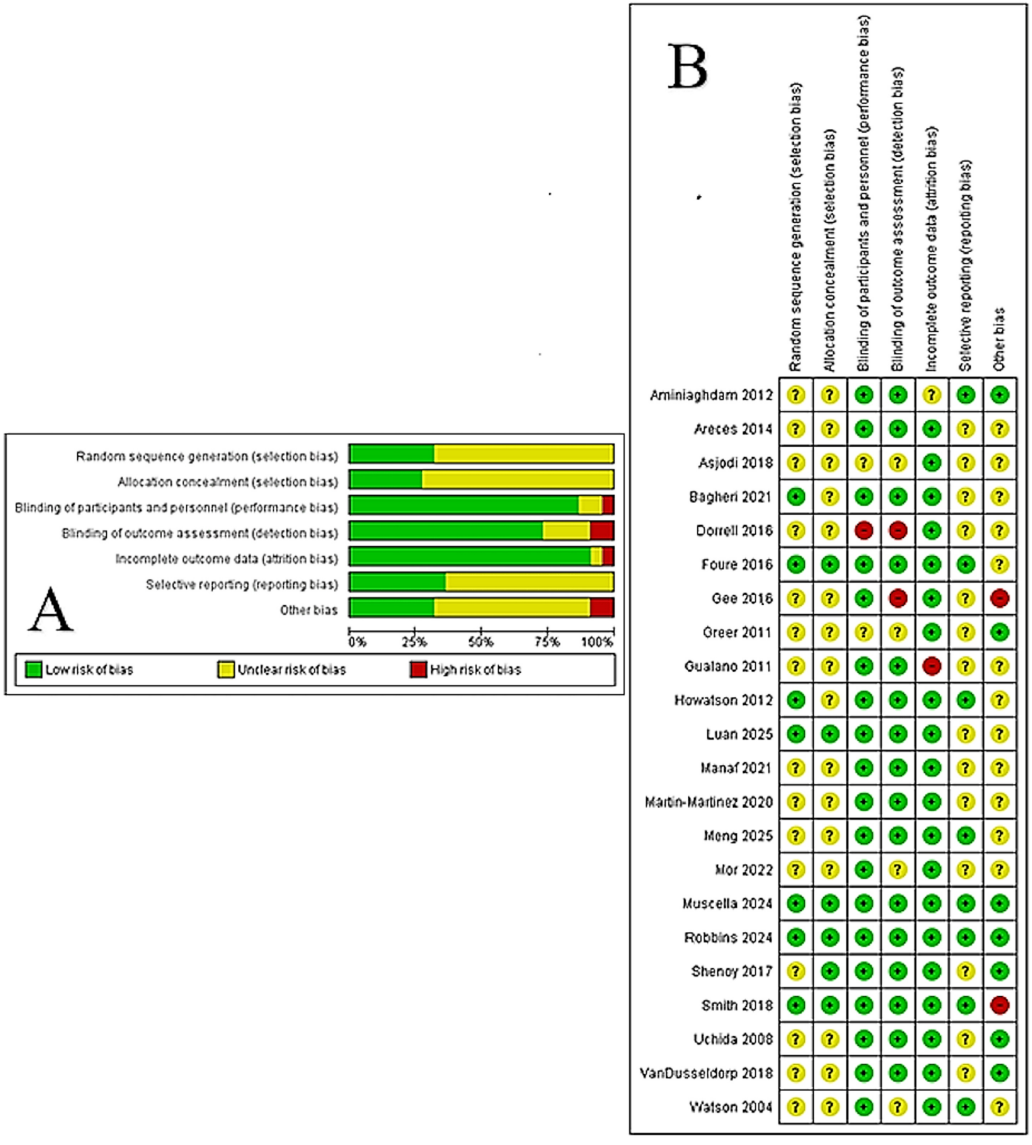
Multiple panels indicating our risk of bias assessment for the included studies (n=22) based on the Cochrane Handbook for Systematic Reviews of Interventions. Green indicates low risk of bias, yellow indicates unclear risk, and red indicates high risk. Panel A shows an overall percentage of risk of bias per bias type. Panel B presents assessment of risk of bias for individual studies. Low risk was the most common level.

Fifteen studies were classified as unclear bias risk due to giving insufficient information on randomization (Figure 3). Sixteen studies did not offer information on allocation concealment. One study was single-blinded (labelled “high risk” as only participants were blinded), and two studies offered limited information on their double-blinded protocol (labelled “unclear risk”). Two did not blind their outcome assessors (high risk), whereas four other studies had unclear risk for detection bias due to insufficient information. One study omitted certain data (high risk of attrition bias), whereas one study gave no participant numbers or information on excluded data (unclear risk). A preliminary study protocol was not available for 15 studies; thus, they were classified as unclear risk of reporting bias. Two studies were labelled as high risk of other bias due to being funded by the supplement producer or failure to control protein intake.

Main Findings

There was substantial heterogeneity amongst the studies regarding the parameters used to measure body composition, and performance (see Table 3). The most common formulation of BCAAs was 2:1:1.

**Table 3:** Summary of significant effects of different doses of BCAAs on body composition and exercise performance * sign marks studies with statistically significant results between BCAA and placebo groups (p<0.05) BCAA: Branched-chain amino acid

Outcome			Percentage of Studies with Significant Studies	Main Findings
Body Composition			1/5 (20%)	Areces et al. [46] (n=46): (5g BCAA) body mass was reduced in both groups with no significant difference (BCAA: -3.0 ± 1.1 and PLAC: - 2.5 ± 1.1 %, P = 0.13)
				Bagheri et al. [48] (n=30): (9g BCAA) body mass, body mass index (BMI), and muscle mass significantly increased in both groups. Body fat percentage significantly decreased in both groups (PLA: -2.7%; BCAA: -3.1%, p<0.05). No significant difference between groups.
				Muscella et al. [59] (n=100)*: 7-10g. BCAA significantly increased muscle mass and fat-free mass in both sexes after six months
				Robbins et al. [60] (n=20): BMI and body weight were not significantly different from placebo after eight weeks of 5g BCAA supplementation
				Howatson et al. [53] (n=12): (20 g BCAA) no significant difference in the calf or thigh circumference between the BCAA and placebo
Exercise Performance	Endurance	Perceived exertion	5/9 (55.5%)	Greer et al. [52] (n=9)*: 48.6g/day BCAA group had significantly lower rates of perceived exertion at 75(-11%), and 90 minutes (-16%) after cycling than the placebo trial (p<0.05).
				Luan et al. [54](n=11)*: 0.2g/kg bodyweight BCAA significantly reduced perceived exertion with lower VAS values in the BCAA cohort
				Manaf et al. [55](n=18)*: approx. 9.4g BCAA/day significantly reduced perceived exertion levels (-3.5%,) in the BCAA group compared to the placebo group, after a cycling time trial
				Muscella et al. [59](n=100)*: 7-10g BCAA significantly reduced fatigue after exercise (women experienced greater reduction in fatigue)
				Robbins et al. [60](n=20)*: 5g BCAA significantly decreased fatigue score (measured with VAS) after 8 weeks of BCAA supplementation
		Maximum repetitions	2/3 (66%)	Asjodi et al. [47] (n=50)*: Approx. 1.5 g BCAA significantly increased maximum repetitions at 72h post-exercise than any other group
				Robbins et al. [60](n=20)*: 5g BCAA significantly increased number of chair stand repetitions after eight weeks of BCAA supplementation
		Time to exhaustion	¼ (25%)	Gualano et al. [31](n=7)*: 300mg/kg BCAA supplementation promoted a greater time to exhaustion (+17.2%) when compared to placebo.
		Cycled distance	0/1 (0%)	Greer et al. [52] (n=9): No significant difference between BCAA and placebo (BCAA: 4.4 ± 0.5km vs PLAC: 3.9 ±0.4 km, p>0.05)
		Cycling efficiency	1/1 (100%)	Luan et al. [54] (n=11)*: 0.2g/kg bodyweight BCAA significantly increased cycling efficiency in BCAA cohort (measured blood respiratory gases)
	Strength	Contraction force and torque	2/7 (29%)	Howatson et al. [53] (n=12)*: 20g BCAA led to a significantly lower decrement in force and greater recovery of force, compared to placebo. 24h after exercise, the peak decrement in force from pre-exercise maximal voluntary contraction was -18% in the BCAA vs -27% in the placebo(p=0.01). (Compared to pre-exercise force)
				Vandusseldorp et al. [64] (n=20)*: Force output(N/m) was significantly reduced after resistance training at all post-exercise time points (0-72h) for placebo, whereas for the BCAA group, the force output was only significantly decreased 0-4hr, becoming non-significant at 24h and onwards.
		Power	5/8 (57%)	Dorrell et al. [49] (n=5)*: Mean power and peak power decreased in both groups after resistance training; however, BCAA supplementation with 6g or 18g decreased the peak power decrement significantly compared to placebo (BCAA 6g: 1107 ± 27 W vs. BCAA 18g: 1133 ± 46 W vs. PLAC: 1044 ± 69 W respectively, p<0.05)
				Manaf et al. [55] (n=18)*: 9.4g BCAA supplementation yielded significantly higher mean power output (+4.6%) after cycling, compared to placebo (BCAA: 130.2 W± 4.5; PLAC: 124.5 ± 4.5, p<0.001)
				Mor et al. [58] (n=24)*: 5g BCAA significantly increased average power after aerobic exercise whereas in the placebo group, power decreased non-significantly. (Average power in BCAA group was 6.5% higher than PLAC group)
				Muscella et al. [59] (n=100)*: 7-10g BCAA significantly increased 1-repetition max power (men experienced greater increase in 1-RM)
				Robbins et al. [60] (n=20)*: 5g BCAA significantly increased handgrip strength (kg)
		Vertical Jump performance	2/6 (33%)	Dorrell et al. [49] (n=5)*: Countermovement jumps, and squat jump distances were reduced after resistance training in all groups; however, BCAA supplementation significantly decreased the performance decrement. Moreover, the decrement in performance was significantly lower with 18g compared to 6g BCAA. CMJ (BCAA 6g:57.7 ± 8.0 cm vs. BCAA 18g: 59.1 ± 7.9 cm vs. CON: 56.6 ± 7.9 cm respectively) and SJ (BCAA 6g: 52.8 ± 9.9 cm vs. BCAA 18g: 54.0 ± 9.9 cm vs. CON: 51.7 ± 10.7 cm respectively)
				Gee et al. [51] (n=11)*: 20 g BCAA significantly decreased the jump height decrement (BCAA: 52.8± 5.9 cm; PLAC: 50.6± 7.3 cm, p=0.031)
		Medicine Ball throw distance	½ (50%)	Gee et al. [51] (n=11)*: The distance the ball was thrown decreased after resistance training in all groups. 20g BCAA supplementation significantly decreased the decrements in distance thrown compared to placebo (4.37± 0.61 m vs 4.22 ± 0.64 m, p=0.044).
	Measures of speed	Time to completion	2/2 (100%)	Manaf et al. [55] (n=18)*: The group supplemented with 9.4g BCAA took significantly less time (-6.7%) to complete a running time trial compared to placebo.
				Robbins et al. [60] (n=20)*: 5g BCAA significantly decreased time to complete 400m walk
		Running pace	0/1 (0%)	Areces et al. [46] (n=46): No significant difference between groups (3.3 ± 0.4 vs. 3.3 ± 0.5 m s^-1^, respectively, 0.98)
	Muscle recovery	Perceived muscle soreness	5/11 (45%)	Dorrell et al. [49] (n=5)*: 6g and 18g BCAA significantly reduced muscle soreness (-9% and 18%, respectively) compared to placebo. Significant difference between 6g and 18g (p=0.025)
				Howatson et al. [53] (n=12)*: 20g BCAA significantly reduced muscle soreness 24h and 48h after plyometric exercise, compared to placebo (at 24h: -33%, at 48h: -23%)
				Meng et al. [57](n=24)*: 40mg/kg BCAA significantly reduced DOMS after exercise at 24h and 48h post-exercise
				Muscella et al. [59](n=100)*: 7-10g BCAA significantly reduced DOMS after exercise (women experienced greater reduction in DOMS)
				Vandusseldorp et al. [64] (n=20)*: approx. 19g BCAA led to significantly lower levels of muscle soreness 48h (-35%) and 72h (-64%) after resistance training, compared to placebo(maltodextrin).
	Measures of fitness	Heart rate	1/4 (25%)	Manaf et al. [55] (n=18)*: Average HR found to be significantly elevated (+1.5%) in the BCAA supplemented group, compared to placebo

Body Composition

Five studies [46,48,53,59,60] investigated the effect of BCAA supplementation on body composition by measuring body mass, BMI, fat-free mass, muscle mass or limb circumference. Only one study had statistically significant results. Muscella et al. [59] found that 100mg/kg bodyweight BCAA supplementation significantly increased muscle mass (BCAA: 2.2±1.3 kg, PLA: -0.6±2.3 kg, p<0.001), and fat-free body mass (BCAA: 1.1±1.2 kg, PLA: -0.5±2.3 kg, p<0.001).

Performance

Parameters that could be broadly categorized as ‘endurance’ included perceived exertion, maximum repetitions, time to exhaustion, cycling distance, and cycling efficiency. Seven out of 10 studies found that BCAA usage significantly improved endurance measures. For example, Manaf et al. [55] found a significant reduction in the perceived exertion after a cycling trial (BCAA: 13.6±0.4 points, PLA: 14.1±0.4 points, p<0.05), whilst Gualano et al. [31] showed that BCAA supplementation significantly increased the time to exhaustion.

Parameters that measured strength included contraction force & torque, power, vertical jump performance, and medicine ball throw distance. Only eight of the 16 studies showed significant improvements in measures of strength. These include the study by Howatson et al. [53], which found a significantly reduced decrement in contraction force after plyometric exercise (BCAA: -18% PLAC: -27%, p=0.01), and that by Manaf et al. [55], which showed a significantly increased power output during cycling (BCAA: 130.2 W± 4.5; PLAC: 124.5 ± 4.5, p<0.001).

Measures of speed included time to completion, and running pace. Manaf et al. [55] found a significant improvement in the time to completion (BCAA: 66 min, PLAC:71 min, p=0.04). Robbins et al. [60] found significantly reduced time to walk 400 m with BCAA supplementation (BCAA: -14%; PLAC: +13%, p<0.001). Areces et al. [46] did not find a significant improvement in running pace.

Certain studies also looked at the impact of BCAAs on recovery, measured by investigating perceived muscle soreness. Five out of 11 studies found a significant reduction in muscle soreness between the BCAA group and the placebo group.

Finally, heart rate was used as a measure of fitness in four studies, with Manaf et al. [55] finding a significant increase in heart rate compared to placebo (BCAA: 151.2±3 BPM, PLA: 148.9±3 BPM, p ≤ 0.001).

Discussion

This systematic review aimed to assess the effect of BCAA supplementation on body composition, and exercise performance. We found limited evidence of a significant BCAA effect on body composition i.e. one study out of five. For performance, we found some studies with significant results with BCAA supplementation (13/22 studies); however, they were inconsistent. Overall, our findings in this review support the hypothesis that BCAA supplementation has an ergogenic effect on recovery outcomes (with moderate certainty evidence). However, there is inconsistent and context-dependent evidence to support the hypothesis that BCAAs have an ergogenic effect on body composition, strength, and endurance performance.

Body Composition

We hypothesized that BCAA supplementation would increase lean muscle mass; however, our findings mostly do not support this hypothesis. The mechanism behind the possible effect of BCAA on body composition is not clear, but BCAAs are thought to increase lean muscle mass by stimulating muscle protein synthesis [35], inhibiting muscle catabolism [36], and stimulating lipid oxidation [31]. Consequently, as free fatty acid oxidation is increased, especially in glycogen-deprived states or fasted states, lean muscle mass is spared, leading to a net increase in lean muscle mass.

Muscella et al. found that 100mg/kg significantly increased muscle mass and fat-free mass after six months of supplementation [59]. The significant results could be due to having a longer supplementation period, and a larger sample size (n=100) compared to other studies with non-significant results [46,48,53,60]. Men had a more pronounced increase in muscle mass gained, whereas women had a more pronounced increase in fat-free mass. The more pronounced difference in males could be due to higher baseline muscle mass, and testosterone levels, which increase the anabolic effect of BCAA. Women show a lower impact from BCAA supplementation on muscle mass, most likely due to hormonal differences and lower baseline muscle mass. This indicates there are sex-specific responses to BCAA supplementation. They suggest that women benefit more from fat oxidation properties and nutrient timing (before and after exercise) of BCAA supplementation, leading to increased lean mass, and aiding weight maintenance rather than weight loss. However, the strength of their conclusions is reduced by the modest sample size (despite being the largest in this review), incomplete lifestyle control, and limited follow-up.

Areces et al. found a decrease in body mass for both groups with no significant difference [46]. The decrease could be attributed to water loss due to the short follow-up period. Hence, the results may have been more accurate if the follow-up period had been longer. Furthermore, Bagheri et al. found an increase in muscle mass and a decrease in fat with no difference between groups [48]. Similarly, Howatson et al. found no difference in limb circumference after BCAA supplementation [53]. Our findings agree with previous literature. Spillane et al. failed to find an ergogenic effect of eight-week BCAA supplementation (9g/day) on muscle mass after resistance exercise [35]. Similarly, Antonio et al. found 18g of EAA (including 9g of BCAA) to have no significant effect on body composition after six weeks of resistance training [66]. However, these studies [35,66] were not randomized controlled trials. Hence, selection bias may have confounded the results, which is why they were not included in this review.

Contrastingly, there are certain studies that disagree with our findings. Dudgeon et al. found BCAA supplementation (26g/day) to preserve lean muscle mass in a 12-week trial carried out with young men on a calorie-restricted “cut” diet [36]. The favorable BCAA effect could be attributed to a longer duration of supplementation and/or a higher dose of BCAAs. Hence, this could indicate that larger BCAA doses may be needed to reach a certain threshold where BCAAs would have a significant effect. However, Dudgeon et al. [36] used a supplement that contained other ingredients such as L-Citrulline, which was shown by Hwang et al. [67] to increase lean mass; hence, it was not included in our review. Moreover, the lack of change in lean muscle mass raises the question about whether the effect can truly be attributed to the BCAA supplement or just poor adherence to the diet.

Moreover, Ikeda et al. investigated the effect of 3.4g BCAA on postmenopausal women, combined with rehabilitative exercise after total hip arthroplasty, and found that the participants had increased upper limb muscle mass [68]. However, the positive effect could be due to age, sex or untrained people having a greater potential for muscle increase [69]. The study by Ikeda et al. was not included as it did not have solely healthy participants [68]. However their significant findings indicate that further research into the effects of BCAAs in post-surgical recovery patients is warranted.

Given the current literature, the evidence for BCAAs improving lean muscle mass is equivocal, especially compared to more complete protein supplements such as whey protein which have been proven to enhance muscle protein synthesis even in calorie-restricted diets [70]. There appear to be some significant results with longer term supplementation in resistance-trained people with sex-specific differences.

Exercise Performance

Thirteen studies had significant findings for exercise performance outcomes. Some findings support the hypothesis that BCAAs have an ergogenic effect on exercise performance, such as increasing endurance, strength, muscle recovery, and speed. However, despite showing positive findings, the mechanism of action is not well established, which contributes to the controversy between studies with significant and non-significant findings.

Endurance

Seven studies found a significant BCAA effect on endurance; however, the evidence is low, and likely context-dependent (environment, glycogen status, exercise modality). Several mechanisms are proposed for how BCAAs may increase endurance. When performing long-term exercise, glycogen stores are depleted, and transamination enzymes in muscles increase their activity to use BCAAs for energy [31,71]. Consequently, it is thought, with BCAA supplementation, there is a higher availability of BCAAs to be used as an energy resource in glycogen-deprived states.

Another theory is based on the effect BCAAs may have on lipid oxidation. Gualano et al. [31] stipulate that the tricarboxylic acid cycle is regulated by the condensation of sufficient amounts of oxaloacetate, and citrate. As glycogen concentrations cannot support the oxaloacetate demands during severe fasting or exhaustive exercise, free fatty acid oxidation is limited by the availability of carbohydrates. Hence, they hypothesized that an increased BCAA availability would lead to increased lipid oxidation which could increase performance. Supporting their hypothesis, Gualano et al. [31] found a significantly reduced respiratory exchange ratio (RER), and better performance in the BCAA group which conveyed increased lipid oxidation. They also suggest the decreased RER indicates a glycogen-sparring effect of BCAA supplementation which could further improve endurance by increasing energy substrate availability [31]. Gualano et al. [31] also suggest that exercise capacity may be improved by BCAA supplementation by reducing the fatiguing effects of hypoglycemia. They found BCAAs to prevent exercise-induced hypoglycemia, especially in glycogen-deprived individuals.

Moreover, endurance may be enhanced by BCAAs decreasing central fatigue. Exercise is associated with increased serotonin in the brain, and elevated central fatigue [33,34]. Previous literature indicates that the concentration of serotonin is proportional to the rate of tryptophan passing through the blood-brain barrier, and BCAAs compete for the same carrier protein that tryptophan uses to reach the blood-brain barrier [72,73]. Consequently, an increased plasma level of BCAA may decrease the amount of tryptophan passing the blood-brain barrier, and, thus, serotonin production [72,73]. However, there have been discrepancies between studies investigating the effect of BCAAs on central fatigue. Manaf et al. [55] suggest that the discrepancy exists due to inter-individual differences such as different rates in TRP transport or sensitivity to serotoninergic activity.

Manaf et al. [55] found perceived exertion to be significantly reduced in the BCAA group. They hypothesize that BCAAs may decrease mental fatigue or alter perceived effort without affecting neuromuscular factors associated with fatigue development. They claim the BCAA effect may be clearer in participants with lower training levels, thus suggesting that the possible ergogenic effects of BCAA may be limited to this group of athletes [55].

Greer et al. [52] suggest that the mechanisms underlying the central fatigue hypothesis take one hour to manifest as they found a significantly lower perceived exertion rate for the BCAA group, 75 and 90 minutes after commencing exercise. This is further supported by Manaf et al. [55], who indicate that BCAA supplementation has shown more favorable effects in “slower” marathon runners, possibly due to longer exercise periods.

Luan et al. have found supplementation with 0.2g/kg bodyweight BCAA to reduce perceived exertion [54]. One mechanism they propose is that BCAAs prevent blood ammonia levels from increasing. Hence, as ammonia causes central fatigue when crossing the blood-brain barrier, BCAAs would alleviate central fatigue. However, the lack of muscle biopsies and short-term nature of their study limit the generalizability of their results.

Muscella et al. found that 100mg/kg BCAA (7-10g for their participants) significantly reduced fatigue levels post-exercise, the effect being more prominent in women [59]. They suggest that women have an enhanced effect, possibly due to hormonal influences on amino acid metabolism, by experiencing increased protein oxidation during the luteal phase of the menstrual cycle and estrogen affecting how BCAAs are utilized during exercise to decrease perceived exertion.

Robbins et al. also found that 5g BCAA reduced perceived exertion in older adults [60]. Like other studies [59,65], they suggest that BCAA reduce fatigue levels through the central serotonin synthesis pathway but also by modulating the kynurenine pathway and reducing neurotoxic metabolites associated with fatigue. However, their results are limited by participant sex imbalances and the absence of biomarker assessment.

Both Areces et al. [46] and Uchida et al. [63] found no significant difference in endurance between BCAAs and placebo. The non-significant effect may be attributed to the lower BCAA doses used (Areces et al.: 5g and Uchida et al: approx. 5.34g) compared to studies with significant effects, which used higher doses (e.g. Manaf et al: 9.4g and Greer et al.: 48.6g). However, this should be considered with caution as Asjodi et al. [47] found significantly increased endurance with lower BCAA doses (approx. 1.5g), yet their participants were untrained which may have amplified the effect. Consequently, future studies should be carried out with higher doses of BCAAs to investigate the dose-response of BCAAs on endurance.

BCAAs are not the sole supplement that are advertised as performance-enhancing; Creatine is another popular pre-workout ergogenic aid used by athletes for various performance gains such as enhanced power, strength, endurance, and muscle recovery [74]. Creatine was studied extensively, and it was found that it works by increasing available ATP in muscles [74]. Even though there is limited comparative analysis between BCAAs and creatine, the evidence supporting creatine’s ergogenic effects is less equivocal and more consistent than that for BCAAs [74]. Mor et al. found both BCAA and creatine to improve anaerobic capacity and endurance [58]. However, despite creatine’s proven ergogenic effects, current literature suggests that the efficacy of creatine supplementation is more limited for endurance sports [74]. Wax et al. found that the ergogenic potential of creatine is diminished as exercise duration increases [74]. Hence, BCAA supplementation may have more pronounced effects during prolonged physical activity, for example, based on Greer et al. [52] trial, longer than one hour. This indicates that further research should be carried out to study BCAA and creatine supplementation in parallel and possibly, even combined, to assess their effects on endurance.

Strength

Eight studies found significant results for strength outcomes, however with low to moderate evidence that BCAAs improve strength recovery with little support for acute strength enhancement. The mechanism for how BCAA supplementation may increase strength is not well understood. It is believed to be a combination of BCAAs stimulating myofibrillar protein synthesis via mTORC1 and p706S kinase [29] and inhibiting muscle catabolism [36], thus accelerating recovery. Consequently, with a more rapid recovery, performance may be regained more quickly, which may allow faster strength development.

Muscella et al. reported that daily BCAA supplementation (7-10 g) over six months significantly improved one-repetition maximum power, with a more pronounced effect observed in male participants [59]. Despite having lower doses than other studies, their significant results could be attributed to having the longest trial period in this review i.e. six months. The difference between sexes could be attributed to menstrual cycle-related hormonal differences, as women experience higher protein catabolism during the luteal phase. This means protein requirements for women fluctuate with the menstrual cycle, which may affect strength gains if protein intake is not adjusted when the requirement increases. However, their conclusions are limited to the 20 to 48-year age group.

Robbins et al. found that 5g BCAAs significantly increased handgrip strength in older participants (60-80 years) [60]. The lower BCAA dose and shorter period of supplementation may have a significant effect (compared to studies with non-significant findings and higher doses) due to the participants being untrained which has been found to be associated with a greater BCAA effect. However, their conclusions are limited by the sample size, type of exercise and age group.

Dorrell et al. found that both 12g and 36g alleviate the decrement in muscle performance after resistance training, in a dose-dependent manner [49]. Their results were similar to those of Howatson et al. [53] which were more prominent. However, the difference in results could be attributed to different factors. Dorrell et al. [49] employed a resistance training design that would emulate real-life exercise, whereas Howatson et al. used a high-intensity resistance training program to maximize muscle damage markers [53]. As da Luz et al. [75] have shown that perceivable effects of prophylactic supplementation, such as BCAAs, are more evident under conditions of greater muscle damage, a more prominent effect was expected in the study by Howatson et al. [53]. Moreover, the more evident effect could be attributed to a higher total dose of BCAAs employed in Howatson et al.(260g) compared to that of Dorrell et al. (12g or 36g). The lower BCAA dose could also explain why Foure et al. [50] did not find a significant difference in strength outcomes. Greer et al. suggest that future studies should also measure blood BCAA levels to find the exact plasma levels at which effects are significant [52].

Like Howatson et al. [53] and Dorrell et al. [49], Vandusseldorp et al. [64] also found BCAA supplementation (approx.19g) to significantly reduce the decrement in muscle strength after exercise. However, it could be argued that their maximal voluntary isometric contraction regimen is not representative of all types of athletic performance movements, and their results may be limited to isometric contractions. Hence, future research should consider exploring the effects of BCAAs on different types of performance movements.

Moreover, like in other similar studies [55,63], Vandusseldorp et al. [64] did not control the participants’ diet completely; hence, the true effect may be confounded by variations in protein intake between participants. Moreover, even though Vandusseldorp et al. controlled the protein intake (1.2g/kg BW/day) and applied an amount lower than the recommended value for resistance training males [76], it could be argued that the dietary protein may have been enough to produce favorable performance outcomes, hence BCAAs may have had a negligible effect.

Another reason why there are inconsistencies in findings could be the level of training the participants possess. Cadore et al. suggest that untrained individuals have a greater anabolic response than well-trained participants [77], which may indicate that the effects of BCAA supplementation may be less evident in well-trained subjects.

Caffeine is another common ingredient in pre-workout supplements that is advertised and proven to increase performance [41]. The mechanisms behind caffeine’s ergogenic effects are still to be elucidated but it is believed to be a combination of recruiting more motor units and reducing fatigue via adenosine inhibition [78]. Like creatine, the evidence for caffeine’s ergogenic effects is stronger than that for BCAAs [6,41]. However, future research should consider a comparative analysis to assess their effects in parallel. Furthermore, one limitation of caffeine is habituation, as its ergogenic aid has been indicated to decrease with long-term use [41]. Consequently, BCAAs should also be assessed for long-term habituation. 

Although BCAAs show some effect in stimulating muscle synthesis, current literature indicates that more complete proteins, including all EAA, offer a higher/maximal muscle protein synthesis response [6]. Marcon et al. argue that athletes who perform regular exercise and have specific protein requirements can achieve the same dietary needs with a balanced diet [6]. Future studies could compare the effect of BCAAs and that of more complete protein supplements in the context of different exercise modalities.

Speed and Fitness

Manaf et al. [55] and Robbins et al. [60] had significant findings for speed and fitness. They found a significantly lower completion time for the BCAA group which could be attributed to lower RPE and increased power. Moreover, Manaf et al. found a significantly higher heart rate for the BCAA group which could show that the BCAA group exercised at a harder intensity compared to the control group, possibly due to lower perceived exertion [55]. However, this is unlike previous studies which found no difference [65] or lower HR [79]. Thus, future research should be performed to assess the effect on HR.

Muscle Recovery

Five studies found significantly lower muscle soreness with BCAA supplementation after exercise [49,53,57,59,64]. However, four have not enforced a strict diet that was 100% monitored, hence variability in protein intake or calories may have confounded the results. These studies have found that BCAA supplementation significantly reduced muscle soreness 24 to 96 hours post-exercise and not immediately after. The level of evidence is moderate, showing more consistent findings, particularly after eccentric/resistance exercise.

The mechanism for aiding muscle recovery may be related to reducing factors that contribute to peripheral fatigue. Vandusseldorp et al. suggest that the increased muscle recovery may be related to BCAA degradation producing glutamine [64]. Research indicates that intense exercise causes increased inflammation. The increased inflammatory response was discovered to increase the sensitivity of muscle nociceptors which is correlated with the elevated feeling of soreness [80]. After consumption, some BCAAs may be transaminated to produce glutamate, later converted into glutamine [11]. Glutamine can be taken up by immune cells as a substrate to decrease the inflammatory response [11]. Nicastro et al. suggest that BCAA supplementation decreases muscle inflammation by increasing the bioavailability of amino acids, especially glutamine, for immune cells [11]. However, this theory should be tested by further studies. Furthermore, BCAAs are be hypothesized to be involved in muscle sarcolemma membrane preservation/repair, yet more research is needed.

Both Gee et al. [51] and Shenoy et al. [61] supplemented participants with 20g BCAAs yet failed to find a significant difference in muscle soreness. However, the soreness scores were lower in the BCAA group. As both studies used trained participants, the results did not reach significance, possibly because the BCAA effect is limited in more trained individuals.

Another factor that may impact the BCAA effect is muscle mass. Shimomura et al. gave 5g BCAA to both women and men and found a significant ergogenic effect on muscle soreness, observing a greater effect in women than men [21]. They explain that women had a lower muscle mass and thus possibly needed less BCAA to produce a significant effect [21]. However, this study should be considered with caution as they employed a crossover design, which is limited by the repeated bout effect. Muscella et al. suggest that men may exhibit a more pronounced reduction in muscle damage markers, lactate, and ammonia (which are associated with fatigue and muscle soreness) due to higher muscle mass and more efficient BCAA absorption compared to women [59].

Timing may also be a key factor in BCAA effect on muscle soreness. Howatson et al. [53] claim that early feeding may increase BCAA efficacy because BCAA blood levels peak 30 minutes after ingestion. Contrastingly, Ra et al. argue that pre-workout supplementation is more efficient as it may limit muscle catabolism during exercise [16]. Consequently, future studies should investigate different timings.

Future Study Improvements

The inconsistency in findings in the current literature can be due to different study design limitations. Many studies utilize BCAA supplements that include other proven ergogenic supplements such as caffeine or carbohydrates that confound the results; hence, future research should aim for standardised pure BCAA formulations. Moreover, the accuracy may be enhanced if less subjective measures of muscle soreness or exertion are used. Also, in the future, aiming for multicenter RCTs, increasing the sample sizes and using more participants of different ages, sex (especially more women), and ethnicities would further improve the study design. Moreover, training outcomes should be monitored for longer periods. Incremental doses combined with blood BCAA levels may more accurately indicate the threshold for BCAA ergogenic aid. Furthermore, increased exercise length may indicate the true effect on central fatigue. Moreover, future studies should employ strict diets and minimize the repeated-bout effect by separating exercise periods appropriately. The impact of different timings, lengths, and participant training levels should also be investigated. Furthermore, more studies should specifically investigate the effect of BCAA supplementation during different stages of the menstrual cycles. Closely monitoring hormones, blood markers of metabolism and muscle damage may also allow better objective correlation with BCAA doses.

Limitations of Our Review

Our review may be limited by the exclusion/inclusion criteria employed, and screening method, as these may have influenced the studies included (publication bias) and conclusions drawn. This could be improved by using several reviewers to establish criteria, independently screen the articles, and cross-reference all data in the end. The reach could also be increased by adding studies in more languages. Moreover, due to the high heterogeneity between studies, we could not objectively assess our hypothesis and significance of our findings through statistical tests; thus, our study is limited by a Type 2 error. This could be improved by increasing the number of studies with similar outcomes. One other limitation is including industry funded research which may sway the results, hence in the future these could be excluded. Although only studies using pure BCAA formulations were included, one study [31] did not specify the precise composition or ratio, limiting interpretability. Future research should ensure transparent reporting of BCAA composition and ratios to allow better comparison between trials and improve reproducibility.

## Conclusions

In this review of 22 RCTs, we show that, according to current literature, there is consistent evidence to moderately support the hypothesis that pure BCAA supplementation decreases muscle soreness and improves recovery following eccentric or resistance exercise. Until more robust evidence emerges, BCAA supplementation should be considered primarily for recovery rather than performance enhancement. We suggest further research into the mechanisms behind BCAAs while using more participants with diverse backgrounds, employing incremental doses, and a strict diet.

## Appendices

**Table 4:** Complete search strategies for all databases and search engines (search date: August 15, 2025)

Database/ Search Engine	Tailored Search Terms	Filters Applied	Date Searched
PubMed	(("branched-chain amino acid*"[Title/Abstract] OR bcaa[Title/Abstract]) AND (exercise[Title/Abstract] OR "exercise tolerance"[Title/Abstract] OR sport*[Title/Abstract] OR endurance[Title/Abstract] OR resistance[Title/Abstract] OR train*[Title/Abstract] OR athlet*[Title/Abstract] OR stamina[Title/Abstract] OR performance[Title/Abstract] OR fatigue[Title/Abstract] OR fitness[Title/Abstract] OR "muscle damage"[Title/Abstract] OR "muscle soreness"[Title/Abstract])) AND (randomized controlled trial[pt] OR randomized[Title/Abstract] OR randomised[Title/Abstract])	English, Portuguese, Human, Adult (≥18 years)	15/08/2025
Ovid Medline	((branched-chain amino acid* or bcaa) and (exercise or "exercise tolerance" or sport* or endurance or resistance or train* or athlet* or stamina or performance or fatigue or fitness or "muscle damage" or "muscle soreness")).ti,ab. and (random*.ti,ab. or randomized controlled trial.pt.)	English, Portuguese, Human, Adult (≥18 years)	15/08/2025
Scopus	TITLE-ABS-KEY ( ( "branched-chain amino acid*" OR bcaa ) AND ( exercise OR "exercise tolerance" OR sport* OR endurance OR resistance OR train* OR athlet* OR stamina OR performance OR fatigue OR fitness OR "muscle damage" OR "muscle soreness" ) ) AND TITLE-ABS-KEY ( random* )	English, Portuguese, Human, Adult (≥18 years)	15/08/2025
Web of Science	TS=(("branched-chain amino acid*" OR bcaa) AND (exercise OR "exercise tolerance" OR sport* OR endurance OR resistance OR train* OR athlet* OR stamina OR performance OR fatigue OR fitness OR "muscle damage" OR "muscle soreness")) AND TS=(random*)	English, Portuguese, Human, Adult (≥18 years)	15/08/2025
Google Scholar	("branched-chain amino acid*" OR bcaa) AND ("exercise performance" OR "exercise tolerance" OR "muscle damage" OR "muscle soreness" OR endurance OR fatigue OR strength) AND ("randomized controlled trial" OR rct OR "randomised controlled trial") AND ("healthy adults" OR "young adults" OR "recreationally active")	English, Portuguese; first 210 results screened manually	15/08/2025
